# Lactational Exposure to Polybrominated Diphenyl Ethers and Its Relation to Early Childhood Anthropometric Measurements

**DOI:** 10.1289/EHP201

**Published:** 2016-05-06

**Authors:** Kate Hoffman, Michelle Mendez, Anna Maria Siega-Riz, Amy H. Herring, Andreas Sjödin, Julie L. Daniels

**Affiliations:** 1 UNC Gillings School of Global Public Health, Chapel Hill, North Carolina, USA; 2 Nicholas School of the Environment, Duke University, Durham, North Carolina, USA; 3 Division for Laboratory Sciences, National Center for Environmental Health, Centers for Disease Control and Prevention, Atlanta, Georgia, USA

## Abstract

**Background:**

Polybrominated diphenyl ethers (PBDEs) are ubiquitous environmental contaminants that may influence growth and development.

**Objective:**

We investigated the association between exposure to PBDEs via breast milk and anthropometric measurements in early childhood.

**Methods:**

The Pregnancy Infection and Nutrition (PIN) Babies studies followed a cohort of North Carolina pregnant women and their children through 36 months of age. Breast milk samples obtained at 3 months postpartum were analyzed for PBDEs. We collected height and weight records from well-baby doctor visits and also measured children during study visits (n = 246 children with > 1,400 anthropometric measurements). We assessed the relationship between breast milk concentrations of five PBDE congeners—BDEs 28, 47, 99, 100, and 153—and child’s weight-for-age, height-for-age, and weight-for-height z-scores (WAZ, HAZ, and WHZ, respectively), adjusting for age; maternal age, race, prepregnancy BMI; parity; smoking during pregnancy; and breastfeeding, and stratifying by sex.

**Results:**

Overall, PBDE exposures via breast milk were not associated with early-life anthropometric measures in the PIN Babies cohort. When stratified by sex, PBDEs in milk were inversely associated with WHZ for boys; however, associations did not follow a consistent pattern across the concentration gradient and were imprecisely estimated. Among girls, PBDEs tended to be associated with increased WHZ except for BDE-153, which was inversely associated with WHZ, though all estimates were imprecisely estimated.

**Conclusions:**

We observed little evidence of associations between early-life PBDE exposures via breast milk and anthropometric measurements overall; however, our results prompt the need for sex-specific investigations in larger cohorts.

**Citation:**

Hoffman K, Mendez M, Siega-Riz AM, Herring AH, Sjödin A, Daniels JL. 2016. Lactational exposure to polybrominated diphenyl ethers and its relation to early childhood anthropometric measurements. Environ Health Perspect 124:1656–1661; http://dx.doi.org/10.1289/EHP201

## Background

Polybrominated diphenyl ethers (PBDEs) have been added to a variety of household products, including electronics, carpets, and the polyurethane foam in furniture, to delay combustion and meet fire safety requirements (Darnerud et al. 2001). PBDEs are not chemically bound to the products to which they are added and leach into the environment over time (Sjödin et al. 2003). Resistant to degradation, PBDEs are persistent in the environment and have become ubiquitous contaminants (Darnerud et al. 2001; Hites 2004). Dust and diet are thought to be the primary sources of exposure to PBDEs in the general population (Fraser et al. 2009; Johnson et al. 2010; Stapleton et al. 2008, 2012; Wu et al. 2007); however, infants are also exposed to PBDEs prenatally and through breastfeeding (Johnson-Restrepo and Kannan 2009; Toms et al. 2009).

Toxicological data indicate that PBDEs disrupt endocrine function (Butt and Stapleton 2013; Ellis-Hutchings et al. 2006; Richardson et al. 2008; Stoker et al. 2004; Szabo et al. 2009; Zhou et al. 2001, 2002). Prenatal and early-life exposures to PBDEs have been shown to affect body weight in early development in animals (Fernie et al. 2006; Gee and Moser 2008; Kim et al. 2009; Kodavanti et al. 2010; Suvorov et al. 2009). The direction of association, however, has not been consistent across studies; some report increases in body weight following exposure to specific congeners (e.g., Suvorov et al. 2009), and others report no association, or decreases in body weight (Kim et al. 2009; Kodavanti et al. 2010). Observed differences by sex, which have been inconsistent, further complicate our understanding of the relationship between PBDEs and early-life growth and adiposity (Fernie et al. 2006; Gee and Moser 2008; Kim et al. 2009; Kodavanti et al. 2010; Suvorov et al. 2009). Few studies have investigated early-life exposure in human populations; however, prenatal PBDE exposures have been inversely associated with birth weight and body mass index (BMI) in newborn infants in some (Chao et al. 2007; Harley et al. 2011; Zhang et al. 2011) but not all studies (Mazdai et al. 2003; Tan et al. 2009). The implications of exposures to PBDEs in infancy on children’s growth remain unknown. Thus, we prospectively investigated the association between early-life exposure to PBDEs through breast milk and early childhood weight-for-age, height-for-age, and weight-for-height in a cohort of central North Carolina children.

## Methods

### Study Sample

The Pregnancy, Infection and Nutrition (PIN) Babies Study began in 2004 to follow the infants born to women who participated in the PIN Pregnancy and Postpartum studies through 3 years of age (Daniels et al. 2010). Women in this study were enrolled from the University of North Carolina (UNC) prenatal care clinics early in pregnancy. Self-administered questionnaires, telephone interviews, and a brief questionnaire at the hospital after delivery were used to collect health and lifestyle information during the pregnancy. Information on postpartum health and lifestyle, as well as children’s development, was collected during three in-home interviews at 3, 12, and 36 months postpartum. Children’s physicians also provided information on growth and development at well-baby doctor visits. All children in the PIN Babies study were singleton births and free from major birth defects (*n* = 585). For these analyses we have further restricted to term infants (> 37 weeks gestation). All study protocols were approved by the Institutional Review Board at the University of North Carolina-Chapel Hill and the Centers for Disease Control and Prevention (CDC), and all mothers provided informed consent.

### Sample Collection

Of the 585 women in the PIN Babies Study, 304 mothers were still lactating and provided breast milk samples at 3 months postpartum. Women providing breast milk samples were similar to those not providing samples with respect to age, but had higher educational attainment (82.2% vs. 71.1% had at least college education). Participants providing samples were also more likely to be white (11.4% or participants with samples were nonwhite whereas 27.6% of participants without milk samples were nonwhite). Details of the breast milk sample collection are available in the study by Daniels et al. (2010). Briefly, lactating women followed written instructions to pump both breasts, gently mix milk, and use a plastic pipette to transfer milk into three 1.5-mL tubes. Women stored the milk in their freezer until a research team picked up the sample during a home data collection visit later the same day. Samples were then transported on ice to –80°C storage freezers until they were analyzed.

### Sample Analysis

Breast milk samples were analyzed for 9 PBDE congeners by the Organic Analytical Toxicology Branch of the National Center for Environmental Health at the CDC using previously described methods (Sjödin et al. 2004). Briefly, milk samples were added to diatomaceous earth packed in a solid-phase extraction cartridge (3 mL) and ^13^C-labeled internal standards were added. Target analytes and lipids were extracted using an automated modular solid-phase extraction system, which dried the sample onto the diatomaceous earth with pressurized nitrogen and eluted analytes and lipids with dichloromethane. Lipid content was determined gravimetrically, and the final analytical determination of PBDEs was performed by gas chromatography/isotope-dilution high-resolution mass spectrometry. Both wet-weight and lipid-normalized concentrations (reported as nanograms per gram of lipid) were produced. Two quality controls and two blank samples were added to each batch of 16 study samples. Quality assurance practices in the laboratory were regularly monitored (Sjödin et al. 2004).

### Anthropometric Measurement

Infant length (the usual method used before 24 months of age) or standing height and weight were recorded by health care professionals during well-baby visits, which are recommended by the American Academy of Pediatrics at 1, 2, 4, 6, 9, 12, 15, 18, 24, 30, and 36 months of age (AAP 2007). Because there was some variability in the frequency and timing of visits, we linked measurements to the child’s age at the time of the visit (reported by the health care provider) or, when age was not recorded, to age calculated using the visit date and the child’s date of birth. If the day of the month was not reported for a visit, we used the 15th of the month in calculation of age. We excluded information for visits conducted before breast milk samples were collected (≤ 3 months postpartum) to ensure that exposure temporally preceded outcomes. At the 36-month home visit, PIN Babies study staff also conducted in-home assessments of weight and standing height, according to the National Health and Nutrition Examination Surveys protocols (CDC 2007).

Weight and length/height of each child were compared to the CDC/National Center for Health Statistics (NCHS) growth charts to calculate sex-standardized weight-for-age, height-for-age, and weight-for-height *z*-scores (WAZ, HAZ, and WHZ, respectively) (Kuczmarski et al. 2002). Measurements that corresponded to *z*-scores > 5 or < –5 were considered implausible and were excluded from analyses (*n* = 33 observations).

### Statistical Analysis

A total of 1,457 WAZ (mean, 5.9; 1–10 per child), 1,414 HAZ (mean, 5.7; 1–13 per child), and 1,432 WHZ (mean, 5.8; 1–10 per child) measurements were available for the 246 children the PIN cohort from 3 through 36 months of age. Measurements were concentrated in the first year (ages 3–12 months, *n* = 843 measurements of 246 children); however, 350 measurements (*n* = 229 children) were made between 12 and 24 months, and 277 (*n* = 192 children) measurements were made after children were 24 months of age. To determine how PBDEs related to WAZ, HAZ, and WHZ in early childhood, we used linear mixed-effects regression models. Mixed-effects models easily accommodate data that are correlated (multiple measurements in each child) with varying numbers of measurements with variable spacing between measurements (Fitzmaurice et al. 2004). Because PBDE concentrations in breast milk are highly correlated, we used separate models to examine the association between quartiles of breast milk levels of each of the five most detected PBDEs (BDEs 28, 47, 99, 100, and 153) and WAZ, HAZ, and WHZ. A summary measure of PBDEs is sometimes calculated; however, because a number of previous studies have shown differences in the biological activity of PBDE congeners, we present results for each congener separately. We included random effects in all models to allow for child-specific variation in both size and growth rates. Because we anticipated that the relationship between age and anthropometric measurements may not be linear, we investigated a linear trend in age at the time of the measurement as well as a quadratic trend for the fixed and random effects. Using a restricted maximum likelihood ratio test, we determined including the quadratic term for random effects improved model fit.

As reported previously for this population, five PBDE congeners—BDEs 28, 47, 99, 100, and 153—were detected in ≥ 91% of samples (Daniels et al. 2010). Milk samples with PBDE values below the limit of detection (LOD) were assigned a concentration of the LOD divided by the square root of 2 for statistical analyses. The four PBDEs congeners infrequently detected [in < 70% of the PIN Babies sample; BDEs 66, 85, 154, 183 (Daniels et al. 2010)] were not evaluated in relation to childhood anthropometric measurements. Because PBDEs are lipophilic, levels in breast milk are often adjusted for lipid content in epidemiologic studies. Although investigators generally agree that adjustment is beneficial, the most appropriate method of adjustment remains unclear (O’Brien et al. 2015; Schisterman et al. 2005). Accordingly, we used multiple methods to account for confounding by the percent lipid in milk samples. First, we conducted analyses using lipid normalized PBDE concentrations. We also included wet-weight concentrations in regression models and included lipid as a covariate. Finally, we conducted analyses of lipid-normalized concentrations with lipid included as a covariate (O’Brien et al. 2015). In general the results were similar using all methods. Here, we present results using lipid-normalized concentrations, which are more commonly presented, and discuss differences observed using other methods of adjustment.

Based on our *a priori* expectation of their association with both PBDE milk concentrations and childhood anthropometric measurements, we adjusted analyses for maternal age (≤ 25 years, 26–30 years, 31–35 years, and ≥ 36 years), race (white vs. nonwhite), smoking status during pregnancy (yes vs. no), prepregnancy BMI (< 25, 25–30, and > 30), parity (0 vs. ≥ 1), and the child’s age at the time of the measurement (continuous age in months and age^2^). We examined the appropriate form (continuous or nominal) for each covariate in relation to the outcomes (form of variables used is reflected in Table 1). We included a time-varying covariate for breastfeeding status at the time of each anthropometric measurement, indicating that the child was currently breastfeeding, recently breastfed (within 2 months), or was no longer breastfeeding. We did not adjust for maternal education or gestational weight gain because these factors were not strongly related to PBDE levels in breast milk and had little impact on the relationships between milk exposure and anthropometric measurements. Because we anticipated that the impact of exposure to PBDEs via lactation may vary with age, we also considered an interaction term between quartiles of PBDEs and age. Interaction terms between PBDE concentrations and age were not statistically significant and did not provide additional insights into the relationship between PBDEs and anthropometric measurements. Thus, we present results for the average effect of PBDE exposure via breast milk from 3 months of age through 36 months of age.

**Table 1:** Selected characteristics of included PIN Babies participants.

Characteristic	*n* (%) or Mean ± SD
Total population	246 (100.0)
Sex
Male	133 (54.1)
Female	113 (45.9)
Maternal race
White	218 (88.6)
Nonwhite	28 (11.4)
Maternal age (years)
≤ 25	33 (13.4)
26–30	82 (33.3)
31–35	100 (40.7)
≥ 36	31 (12.6)
Maternal education
Less than college graduate	39 (15.9)
College graduate	88 (35.8)
Graduate training or degree	119 (48.4)
Smoking during pregnancy
Yes	9 ( 3.7)
No	232 ( 94.3)
Missing	5 ( 2.0)
Parity
0	130 (52.9)
≥ 1	116 (47.2)
Breast feeding duration
< 10 months	88 (35.8)
10–14 months	113 (45.9)
≥ 15 months	45 (18.3)
Maternal prepregnancy BMI (kg/m^2^)	23.4 ± 4.7
Child WAZ^*a*^	0.1 ± 1.1
Child HAZ^*b*^	0.5 ± 1.0
Child WHZ^*c*^	–0.1 ± 1.2
^***a***^1,457 observations for 239 participants (measured between 1 and 13 times). ^***b***^1,414 observations for 240 participants (measured between 1 and 10 times). ^***c***^1,432 observations for 239 participants (measured between 1 and 10 times).	

We hypothesized that the potential pathway by which PBDE exposures may be associated with growth is through endocrine disruption, which suggests the possibility of differential associations by sex. We conducted sex-stratified analyses to investigate differences in associations between boys and girls. All analyses were conducted in SAS (version 9.2; SAS Institute Inc., Cary, NC).

## Results

Of the children whose mothers provided milk samples (*n* = 304), 266 (87.5%) had height or weight measurements (children with measurements did not differ from other children with respect to included covariates). We excluded children who were born < 37 weeks gestation (*n* = 20). Our final sample consisted of 246 children with at least 1 measurement of height or weight and a maximum of 13 measurements (> 1,400 measurements for each outcome; median measurements per child, 6.0; interquartile range, 4–8 measurements). As in the larger PIN Babies cohort, mothers tended to be > 31 years of age at the time of their child’s birth (52.6%) and were predominantly white (88.6%; Table 1). Children in our sample were slightly more likely to be male (54.3%) and were generally breastfed longer than 10 months (64.2%). WAZ and HAZ for children in our sample were slightly higher at a given age than the 2000 CDC normative population (mean WAZ, 0.1; mean HAZ, 0.5). However, children’s WHZ tended to be lower, indicating that PIN Babies participants were slightly leaner than the normative population.

As reported previously in the PIN babies cohort, BDEs 28, 47, 99, 100, and 153 were detected frequently in breast milk samples (≥ 90.7% of samples; Table 2) and were generally correlated with each other (Daniels et al. 2010). Levels of BDE-47 tended to be higher than those of the other congeners (median breast milk level, 26.8 ng/g lipid), and the levels of BDE-28 were generally the lowest (median breast milk level, 2.0 ng/g lipid).

**Table 2:** Lipid-adjusted concentrations (ng/g lipid) of commonly detected PBDEs in 246 breast milk samples collected at 3 months postpartum in 2004–2006.

PBDE congener	Percent detect (LOD)	Mean	Quartile ranges			
			Q1	Q2	Q3	Q4
BDE-28	97.1 (0.3)	3.2	ND to 1.3	1.4 to 2.0	2.1 to 3.7	3.8 to 49.6
BDE-47	99.6 (1.3)	50.2	ND to 15.7	15.8 to 27.7	27.8 to 54.2	54.3 to 1,430
BDE-99	90.7 (1.1)	10.4	ND to 2.65	2.8 to 5.2	5.3 to 11.2	11.3 to 299
BDE-100	98.4 (0.3)	10.4	ND to 2.4	2.5 to 5.15	5.25 to 10.4	10.5 to 188
BDE-153	99.2 (0.3)	14.8	ND to 2.65	2.8 to 5.6	5.7 to 13.5	13.55 to 229
ND (nondetectable) values are treated as the LOD divided by the square root of 2 in the calculation of the mean.						

**Figure 1:**
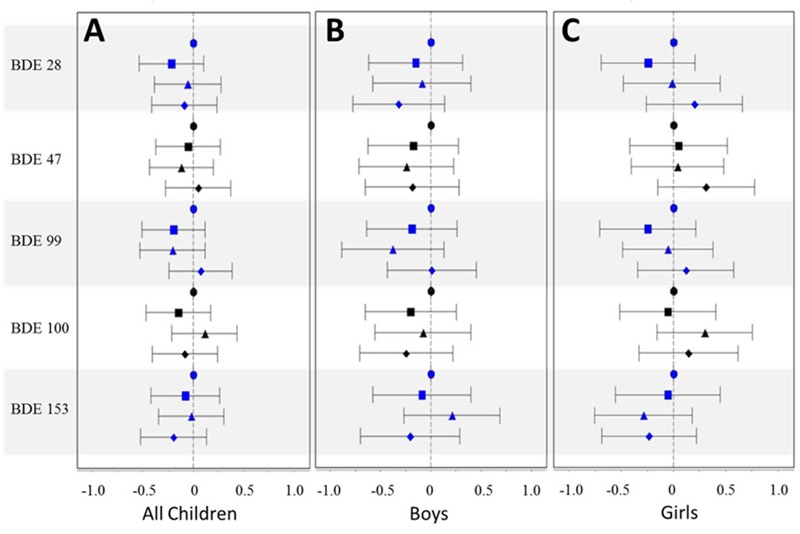
Mean difference estimates and 95% CIs by quartile of PBDE congener [1st quartile (referent) = circle; 2nd quartile = square; 3rd quartile = triangle; 4th quartile = diamond] for WAZ, adjusted for age (age and age^2^), maternal age (≤ 25 years, 26–30 years, 31–35 years, and ≥ 36 years), race (white vs. nonwhite), prepregnancy BMI (< 25, 25–30, and > 30), parity (0 vs. ≥ 1), smoking during pregnancy (yes vs. no), and breastfeeding status (current, within the last 2 months, and no longer breastfeeding): (*A*) full cohort; (*B*) boys only; (*C*) girls only.

**Figure 2:**
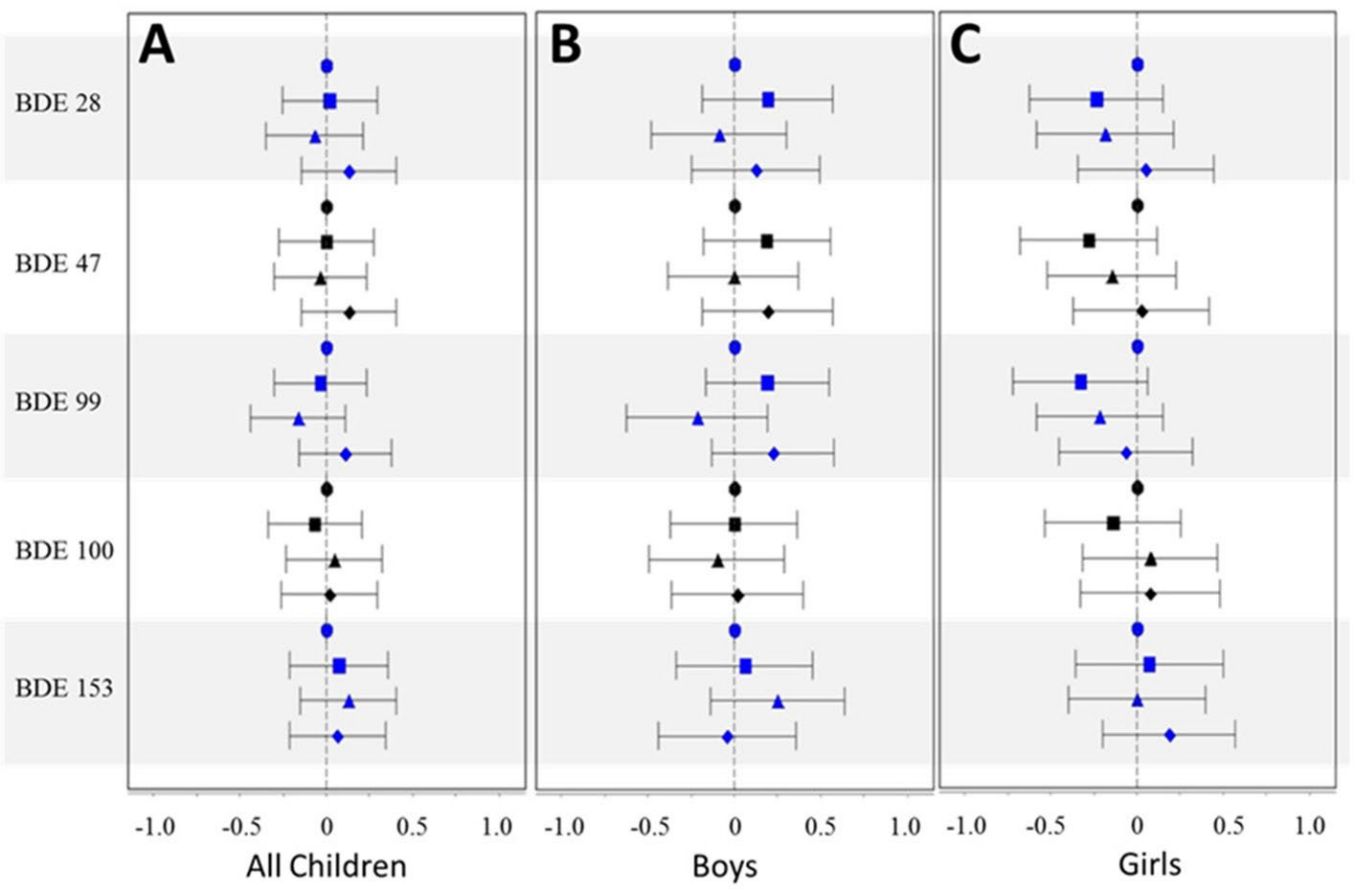
Mean difference estimates and 95% CIs by quartile of PBDE congener [1st quartile (referent) = circle; 2nd quartile = square; 3rd quartile = triangle; 4th quartile = diamond] for HAZ, adjusted for age (age and age^2^), maternal age (≤ 25 years, 26–30 years, 31–35 years, and ≥ 36 years), race (white vs. nonwhite), prepregnancy BMI (< 25, 25–30, and > 30), parity (0 vs. ≥ 1), smoking during pregnancy (yes vs. no), and breastfeeding status (current, within the last 2 months, and no longer breastfeeding): (*A*) full cohort; (*B*) boys only; (*C*) girls only.

**Figure 3:**
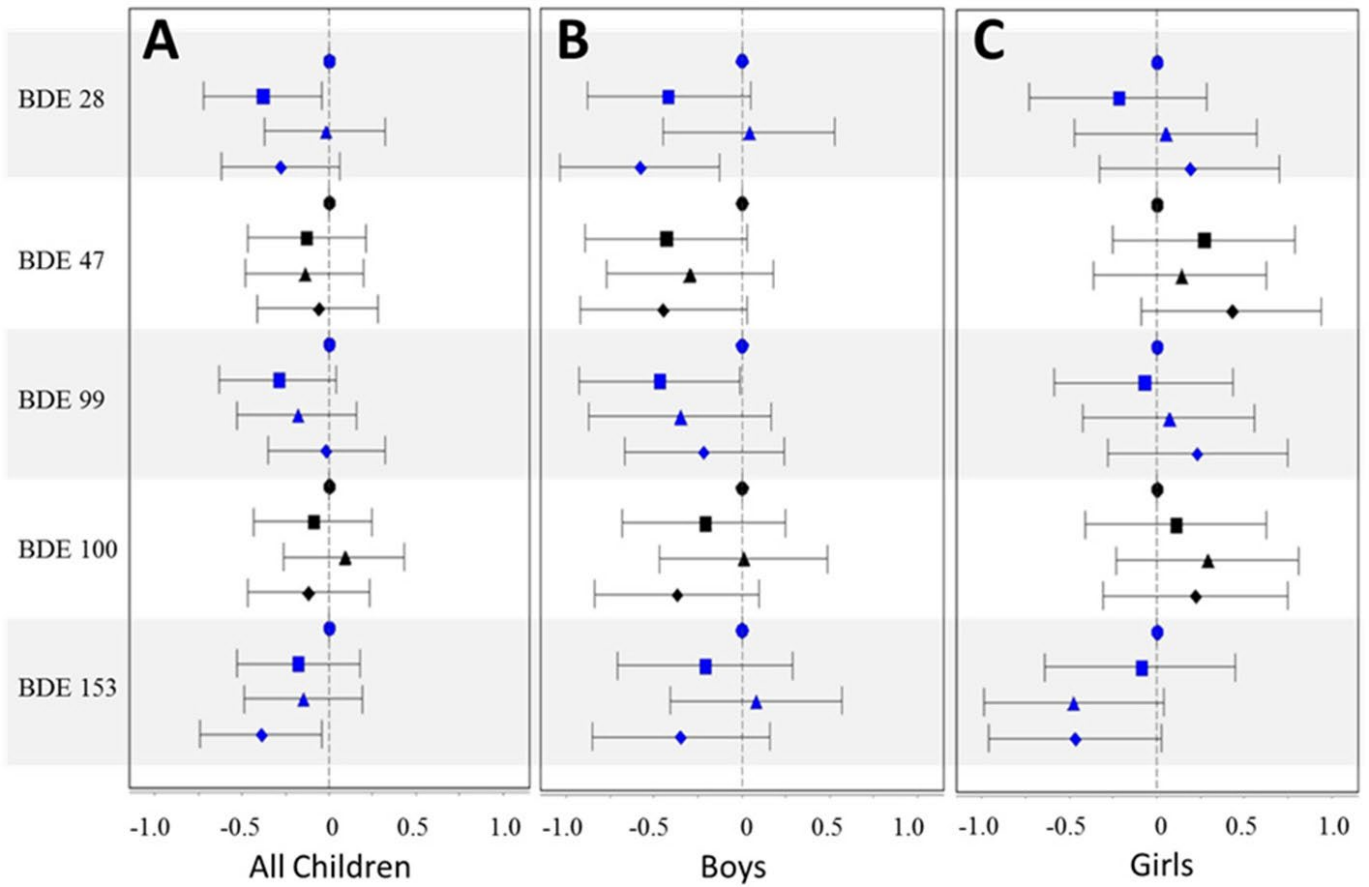
Mean difference estimates and 95% CIs by quartile of PBDE congener [1st quartile (referent) = circle; 2nd quartile = square; 3rd quartile = triangle; 4th quartile = diamond] for WHZ, adjusted for age (age and age^2^), maternal age (≤ 25 years, 26–30 years, 31–35 years, and ≥ 36 years), race (white vs. nonwhite), prepregnancy BMI (< 25, 25–30, and ≥ 30), parity (0 vs. ≥ 1), smoking during pregnancy (yes vs. no), and breastfeeding status (current, within the last 2 months, and no longer breastfeeding): (*A*) full cohort; (*B*) boys only; (*C*) girls only.

Associations between lipid-normalized PBDE levels in breast milk and WAZ, HAZ, and WHZ are shown in Figures 1, 2, and 3, respectively [effect estimates and 95% confidence intervals (CIs) presented in Tables S1–S3]. PBDE exposures via breast milk were generally not associated with early-life anthropometric measures (WAZ, HAZ, and WHZ) in the PIN Babies cohort as a whole, after adjustment for age, maternal age, race, prepregnancy BMI, parity, smoking during pregnancy, and breastfeeding status. Effect estimates generally hovered around the null, did not follow a consistent pattern across the concentration gradient, and were imprecisely estimated. Different methods of adjusting for the lipid content of milk (e.g., including lipid as a covariate) produced slightly different results; however, results with these methods remained largely null and did not provide additional insights regarding the relationship between locational exposure to PBDEs and anthropometric measures in early childhood.

When we stratified by sex, higher levels of lipid-normalized PBDEs in milk tended to be associated with decreased WHZ for boys, particularly for BDEs 28, 47, and 100; however, as in the full cohort (boys and girls combined), the patterns were inconsistent across the concentration gradient and imprecisely estimated and generally not statistically significant (Figure 3B). Results for WAZ were similar, with high PBDE levels in breast milk generally associated with lower WAZ in boys, after comparing all quartiles of exposure to the lowest quartile; but the results were not completely consistent and imprecise (Figure 1B). Associations between lipid-normalized PBDE levels and anthropometric measurements were largely null for girls except for BDE-153, which our data suggest may be inversely associated with WAZ and WHZ [Figures 1C and 2C; mean difference (95% CI) = –0.47 (–0.96, 0.03) and –0.24 (–0.69, 0.22) comparing the 4th quartile to the 1st, respectively]. When we adjusted for lipids by adding a term for lipid to the regression model, effect estimates were further attenuated, with two notable exceptions: First, the inverse association between the highest quartile of BDE-153 and WHZ among girls was unchanged [mean differences comparing the 4th quartile to the 1st, 0.46 (95% CI: –0.98, 0.07)]. Second, the association between BDE-28 and WHZ among girls was more prominently positive [mean difference 2nd quartile, 0.69 (95% CI: 0.19, 1.22); mean difference 3rd quartile, 0.21 (95% CI: –0.32, 0.75); mean difference 4th quartile, 0.68 (95% CI: 0.06, 1.31)].

## Discussion

Despite widespread exposure to PBDEs, relatively little is known about their potential to affect health, particularly regarding children’s growth. In our cohort of central North Carolina children, higher PBDE levels in breast milk were generally not associated with anthropometric measurements from 3 through 36 months of age. There was some suggestion of decreased WHZ in boys more highly exposed via breast milk and increased WHZ in girls; however our small sample hindered our ability to more precisely assess differences in the associations by sex. Although not a direct measurement of adiposity, WHZ is more reflective of fat mass than WAZ or HAZ.

Several previous toxicological studies have documented the endocrine-disrupting properties of PBDEs and reported sex differences in offspring weight gain in the early postnatal period, although the direction of reported associations has been inconsistent. For example, prenatal exposure to the commercial pentaPBDE mixture, DE-71, was associated with decreased weight gain in female but not male rat pups (Kodavanti et al. 2010). Conversely, prenatal exposure to BDE-209 was related to lower weight gain in early development in male rat pups but not female rat pups (Kim et al. 2009)—a finding consistent with our results in boys, which suggested lower WHZ with increasing PBDE levels in breast milk.

The only other study to assess postnatal exposure to PBDE found an inverse association between serum BDE-153 levels and BMI percentile at 7 years of age (Erkin-Cakmak et al. 2015), a finding consistent with our results suggesting lactational exposure to BDE-153 may be associated with lower WHZ. Additionally, previous studies have reported prenatal exposures to be associated with lower birth weight and BMI in newborn infants (Chao et al. 2007; Harley et al. 2011; Zhang et al. 2011). Our data for BDE-153 and WHZ are consistent with these results; however, we did not consistently observe similar associations for other congeners. There are several possible explanations for differences in the results of our work and those from previous research. A primary difference is that our results are based on postnatal PBDE exposure through breast milk, which is correlated with but different from the prenatal exposure metrics used by most previous studies. We did not have exposure data to explore associations reflecting the prenatal period, which may be a more sensitive developmental stage for PBDEs exposure. It is possible that the associations between PBDEs and children’s height and weight differs over time. We did not see differences by age when including an interaction term in models; however, the children in our study were relatively young, and most of the anthropometric measurements were collected in the first year of life, potentially limiting our ability to detect differences through 3 years of age (results not shown).

PBDE milk concentrations in our cohort were higher than concentrations reported by most similarly timed studies from the United States and considerable higher than those reported abroad (Daniels et al. 2010; Erdoğrul et al. 2004; Frederiksen et al. 2009; Johnson-Restrepo et al. 2007; Schecter et al. 2003; She et al. 2007; Toms et al. 2007). Although a clear trend has yet to be established, exposure to PBDEs is likely to decrease in the coming decades because their use has been phased out in many countries.

Our work should be interpreted in the context of several limitations. PBDEs are ubiquitous contaminants in the home environment (e.g., Stapleton et al. 2009), raising the possibility that breast milk samples, which were collected by mothers in their own homes, may been contaminated. Although mothers were given instruction on sample collection to reduce this possibility, exposure misclassification is possible and may have biased results toward the null. Similarly, we have not accounted for competing environmental exposures or dietary patterns in these analyses. However, by design, all children included in analyses were breastfed through 3 months of age, and most were breastfed > 10 months. Although we had > 1,400 anthropometric measurements, our samples size of 246 children was relatively small, limiting our ability to investigate difference in effect by sex or over time. Additionally, generalizability of our results is limited by the homogeneity of the PIN Babies cohort. PIN Babies included mothers that attended UNC prenatal care clinics, delivered generally healthy singleton infants, completed several years of follow-up, and were not very demographically diverse. Mothers in the PIN Babies cohort were highly educated. These mothers may have been more likely to intervene to mitigate suboptimal growth, potentially masking any impact of early-life PBDE exposures. Finally, most of the height and weight measurements for the PIN Babies cohort were collected at doctors’ offices. These measurements may be subject to random error because they were collected at multiple medical office sites by staff members who had not been trained using a standard method. Such error would likely diminish our ability to detect associations. However, we found excellent correlation between the health providers’ measurements and our standardized study measurements; among 50 participants with both measures collected within a 2-week period around 36 months of age, the correlation between the two measurements was 0.85 (*p* < 0.001 for correlation). This comparison offers assurance that health provider measurements provide reliable measures of growth between study visits and contribute favorably to our analysis. Additionally, we benefitted from ability to evaluate this association in a well-characterized, somewhat homogenous, group of mothers and children with reasonable exposure variability.

## Conclusions

We observed little evidence of associations between early-life PBDE exposures via breast milk and anthropometric measurements; however, our results suggesting possible differences in effects between boys and girls prompt the need for sex-specific investigations in larger cohorts. Further study is needed to determine whether PBDEs are adversely associated with children’s growth and whether that trend persists throughout as children age, particularly because altered growth in early life has long-term health implications.

## Supplemental Material

(116 KB) PDF
