# Supplementary material for: Lactational Exposure to Polybrominated Diphenyl Ethers and Its Relation to Early Childhood Anthropometric Measurements
Source: Environ Health Perspect. 2016 May 6;124(10):1656–61. doi: 10.1289/EHP201 (PMC5047775; doi:10.1289/EHP201)
Supplement: (116 KB) PDF [file EHP201.s001.acco.pdf]

Environ Health Perspect
DOI: 10.1289/EHP201

Note to readers with disabilities: *EHP* strives to ensure that all journal content is accessible to all readers. However, some figures and Supplemental Material published in *EHP* articles may not conform to 508 standards due to the complexity of the information being presented. If you need assistance accessing journal content, please contact ehp508@niehs.nih.gov. Our staff will work with you to assess and meet your accessibility needs within 3 working days.

Supplemental Material

Lactational Exposure to Polybrominated Diphenyl Ethers and Its Relation to Early Childhood Anthropometric Measurements

Kate Hoffman, Michelle Mendez, Anna Maria Siega-Riz, Amy H. Herring, Andreas Sjödin, and Julie L. Daniels

Table S1: Mean difference estimates and 95% confidence intervals by quartile of PBDE congener for WAZ, adjusted for adjusting for age (age and age^2), maternal age (<=25 years, 26-30 years, 31-35 years, and ≥36 years), race (white vs. non-white), pre-pregnancy BMI (<25, 25-30, and ≥30), parity (0 vs. 1+), smoking during pregnancy (yes vs. no), and breastfeeding status (current, within the last 2 months, and no longer breastfeeding).

Table S2: Mean difference estimates and 95% confidence intervals by quartile of PBDE congener for HAZ, adjusted for adjusting for age (age and age^2), maternal age (<=25 years, 26-30 years, 31-35 years, and ≥36 years), race (white vs. non-white), pre-pregnancy BMI (<25, 25-30, and ≥30), parity (0 vs. 1+), smoking during pregnancy (yes vs. no), and breastfeeding status (current, within the last 2 months, and no longer breastfeeding).

Table S3: Mean difference estimates and 95% confidence intervals by quartile of PBDE congener for WHZ, adjusted for adjusting for age (age and age^2), maternal age (<=25 years, 26-30 years, 31-35 years, and ≥36 years), race (white vs. non-white), pre-pregnancy BMI (<25, 25-30, and ≥30), parity (0 vs. 1+), smoking during pregnancy (yes vs. no), and breastfeeding status (current, within the last 2 months, and no longer breastfeeding).

Table S1: Mean difference estimates and 95% confidence intervals by quartile of PBDE congener for WAZ, adjusted for adjusting for age (age and age^2), maternal age (<=25 years, 26-30 years, 31-35 years, and ≥36 years), race (white vs. non-white), pre-pregnancy BMI (<25, 25-30, and ≥30), parity (0 vs. 1+), smoking during pregnancy (yes vs. no), and breastfeeding status (current, within the last 2 months, and no longer breastfeeding).

		WAZ		
		**Combined**	**Males**	**Females**
BDE 28	<25th	Reference	Reference	Reference
	25–50th	-0.22 (-0.54, 0.11)	-0.15 (-0.62, 0.31)	-0.24 (-0.69, 0.21)
	50–75th	-0.06 (-0.39, 0.27)	-0.09 (-0.58, 0.40)	-0.02 (-0.48, 0.45)
	>75th	-0.09 (-0.41, 0.23)	-0.32 (-0.78, 0.14)	0.20 (-0.26, 0.66)
BDE 47	<25th	Reference	Reference	Reference
	25–50th	-0.05 (-0.37, 0.27)	-0.18 (-0.63, 0.28)	0.05 (-0.42, 0.52)
	50–75th	-0.12 (-0.44, 0.20)	-0.24 (-0.71, 0.22)	0.04 (-0.40, 0.48)
	>75th	0.05 (-0.27, 0.37)	-0.18 (-0.65, 0.28)	0.31 (-0.15, 0.77)
BDE 99	<25th	Reference	Reference	Reference
	25–50th	-0.20 (-0.51, 0.12)	-0.19 (-0.64, 0.26)	-0.25 (-0.71, 0.21)
	50–75th	-0.20 (-0.53, 0.12)	-0.38 (-0.88, 0.13)	-0.05 (-0.49, 0.38)
	>75th	0.07 (-0.24, 0.39)	0.01 (-0.43, 0.45)	0.12 (-0.34, 0.58)
BDE 100	<25th	Reference	Reference	Reference
	25–50th	-0.15 (-0.47, 0.17)	-0.20 (-0.65, 0.25)	-0.06 (-0.52, 0.40)
	50–75th	0.11 (-0.21, 0.44)	-0.08 (-0.56, 0.40)	0.30 (-0.16, 0.75)
	>75th	-0.08 (-0.41, 0.24)	-0.25 (-0.71, 0.22)	0.14 (-0.33, 0.62)
BDE 153	<25th	Reference	Reference	Reference
	25–50th	-0.08 (-0.42, 0.26)	-0.09 (-0.57, 0.40)	-0.05 (-0.55, 0.45)
	50–75th	-0.02 (-0.34, 0.3)	0.21 (-0.27, 0.69)	-0.29 (-0.76, 0.18)
	>75th	-0.20 (-0.52, 0.13)	-0.21 (-0.70, 0.29)	-0.24 (-0.69, 0.22)

Table S2: Mean difference estimates and 95% confidence intervals by quartile of PBDE congener for HAZ, adjusted for adjusting for age (age and age^2), maternal age (<=25 years, 26-30 years, 31-35 years, and ≥36 years), race (white vs. non-white), pre-pregnancy BMI (<25, 25-30, and ≥30), parity (0 vs. 1+), smoking during pregnancy (yes vs. no), and breastfeeding status (current, within the last 2 months, and no longer breastfeeding).

		HAZ		
		**Combined**	**Males**	**Females**
BDE 28	<25th	Reference	Reference	Reference
	25–50th	0.02 (-0.26, 0.29)	0.19 (-0.19, 0.57)	-0.24 (-0.62, 0.15)
	50–75th	-0.07 (-0.35, 0.21)	-0.09 (-0.48, 0.30)	-0.19 (-0.59, 0.21)
	>75th	0.13 (-0.14, 0.41)	0.12 (-0.25, 0.49)	0.05 (-0.34, 0.44)
BDE 47	<25th	Reference	Reference	Reference
	25–50th	0 (-0.27, 0.27)	0.19 (-0.18, 0.55)	-0.28 (-0.68, 0.12)
	50–75th	-0.04 (-0.31, 0.23)	0.00 (-0.38, 0.37)	-0.15 (-0.52, 0.23)
	>75th	0.13 (-0.14, 0.41)	0.19 (-0.18, 0.57)	0.03 (-0.37, 0.42)
BDE 99	<25th	Reference	Reference	Reference
	25–50th	-0.03 (-0.3, 0.23)	0.19 (-0.17, 0.55)	-0.33 (-0.72, 0.06)
	50–75th	-0.16 (-0.44, 0.11)	-0.21 (-0.62, 0.19)	-0.22 (-0.59, 0.15)
	>75th	0.11 (-0.16, 0.38)	0.22 (-0.13, 0.58)	-0.07 (-0.46, 0.32)
BDE 100	<25th	Reference	Reference	Reference
	25–50th	-0.07 (-0.34, 0.2)	0.00 (-0.37, 0.37)	-0.14 (-0.54, 0.25)
	50–75th	0.04 (-0.23, 0.32)	-0.10 (-0.49, 0.29)	0.08 (-0.31, 0.47)
	>75th	0.02 (-0.26, 0.30)	0.02 (-0.36, 0.40)	0.08 (-0.33, 0.48)
BDE 153	<25th	Reference	Reference	Reference
	25–50th	0.07 (-0.21, 0.36)	0.06 (-0.33, 0.45)	0.07 (-0.36, 0.50)
	50–75th	0.13 (-0.15, 0.40)	0.25 (-0.14, 0.64)	0.00 (-0.40, 0.40)
	>75th	0.06 (-0.21, 0.34)	-0.04 (-0.44, 0.35)	0.19 (-0.20, 0.57)

Table S3: Mean difference estimates and 95% confidence intervals by quartile of PBDE congener for WHZ, adjusted for adjusting for age (age and age^2), maternal age (<=25 years, 26-30 years, 31-35 years, and ≥36 years), race (white vs. non-white), pre-pregnancy BMI (<25, 25-30, and ≥30), parity (0 vs. 1+), smoking during pregnancy (yes vs. no), and breastfeeding status (current, within the last 2 months, and no longer breastfeeding).

		WHZ		
		**Combined**	**Males**	**Females**
BDE 28	<25th	Reference	Reference	Reference
	25–50th	-0.38 (-0.72, -0.04)	-0.42 (-0.88, 0.05)	-0.22 (-0.73, 0.29)
	50–75th	-0.02 (-0.37, 0.32)	0.04 (-0.45, 0.53)	0.05 (-0.47, 0.57)
	>75th	-0.28 (-0.62, 0.06)	-0.58 (-1.04, -0.13)	0.19 (-0.33, 0.70)
BDE 47	<25th	Reference	Reference	Reference
	25–50th	-0.13 (-0.47, 0.21)	-0.43 (-0.89, 0.03)	0.27 (-0.25, 0.79)
	50–75th	-0.14 (-0.48, 0.20)	-0.30 (-0.77, 0.18)	0.14 (-0.36, 0.63)
	>75th	-0.06 (-0.41, 0.28)	-0.45 (-0.92, 0.03)	0.43 (-0.09, 0.94)
BDE 99	<25th	Reference	Reference	Reference
	25–50th	-0.29 (-0.63, 0.04)	-0.47 (-0.93, -0.01)	-0.07 (-0.59, 0.44)
	50–75th	-0.18 (-0.53, 0.16)	-0.35 (-0.87, 0.17)	0.07 (-0.42, 0.56)
	>75th	-0.02 (-0.35, 0.32)	-0.22 (-0.67, 0.24)	0.23 (-0.28, 0.75)
BDE 100	<25th	Reference	Reference	Reference
	25–50th	-0.09 (-0.43, 0.25)	-0.21 (-0.68, 0.25)	0.11 (-0.41, 0.63)
	50–75th	0.09 (-0.26, 0.43)	0.01 (-0.47, 0.49)	0.29 (-0.23, 0.81)
	>75th	-0.12 (-0.47, 0.23)	-0.37 (-0.84, 0.10)	0.22 (-0.31, 0.75)
BDE 153	<25th	Reference	Reference	Reference
	25–50th	-0.18 (-0.53, 0.18)	-0.21 (-0.71, 0.29)	-0.09 (-0.64, 0.45)
	50–75th	-0.15 (-0.49, 0.19)	0.08 (-0.41, 0.57)	-0.48 (-0.99, 0.04)
	>75th	-0.39 (-0.74, -0.04)	-0.35 (-0.85, 0.16)	-0.47 (-0.96, 0.03)
